# “I’m not a teenager, I’m 22. Why can’t I snap out of it?”: a qualitative exploration of seeking help for a first-episode eating disorder during emerging adulthood

**DOI:** 10.1186/s40337-020-00320-5

**Published:** 2020-09-03

**Authors:** Rachel Potterton, Amelia Austin, Karina Allen, Vanessa Lawrence, Ulrike Schmidt

**Affiliations:** 1 King’s College London, Institute of Psychiatry, Psychology and Neuroscience, Section of Eating Disorders, London, UK; 2 The Eating Disorders Service, Maudsley Hospital, South London and Maudsley NHS Foundation Trust, London, UK; 3 King’s College London, Institute of Psychiatry, Psychology and Neuroscience, Department of Health Services and Population Research, London, UK

**Keywords:** Eating disorders, Bulimia nervosa, Anorexia nervosa, Help-seeking, Emerging adulthood

## Abstract

**Background:**

Eating disorders (EDs) typically have their onset during adolescence or the transition to adulthood. Emerging adulthood (~ 18–25 years) is a developmental phase which conceptually overlaps with adolescence but also has unique characteristics (e.g. increased independence). Emerging adults tend to come to ED services later in illness than adolescents, and emerging adulthood’s unique characteristics may contribute to such delays**.**

**Objective:**

This study aimed to explore attitudes towards ED symptoms, and their implications for help-seeking, amongst emerging adults receiving ED treatment through FREED, an early intervention care pathway.

**Method:**

Participants were 14 emerging adults (mean age 20.9 years; SD = 2.0), all currently receiving specialist treatment for a first-episode, recent-onset (< 3 years) ED. Semi-structured interviews relating to experiences of help-seeking were conducted, and data were analysed thematically.

**Results:**

Symptom egosyntonicity, gradual reappraisal and feelings of exclusion from ED discourse were key attitudinal phases prior to help-seeking, each of which had distinct implications for help-seeking.

**Conclusions:**

Emerging adults with first-episode EDs show a distinct set of help-seeking-related challenges and opportunities (e.g. help-seeking for others; help-seeking at transitions; self-sufficiency). This research might be used to inform the development and evaluation of interventions which aim to facilitate help-seeking amongst emerging adults with first-episode recent-onset EDs.

## Plain English summary

Eating disorders (EDs) are serious mental illnesses, often experienced for the first time during adolescence or transition to adulthood. Emerging adulthood is the life-stage between 18 and 25 years and is somewhat similar to adolescence, but also has unique characteristics (e.g. increased independence). Research indicates that emerging adults wait longer than adolescents before seeking help for ED symptoms. This study aimed to explore reasons for delayed help-seeking amongst emerging adults. We interviewed 14 emerging adults current receiving treatment for an ED through an early intervention care pathway, finding a consistent set of themes and experiences among them. Early in their ED, emerging adults tended to interpret their symptoms positively and did not want help tackling these symptoms. Whilst emerging adults gradually reappraised their symptoms, some participants believed that EDs were only possible for those with extreme low-weight and only affect “white teenage girls”. These beliefs contributed to reluctance to seek help amongst those who did not fit that description. Policy aimed at quickly connecting emerging adults with professionals might usefully focus on tackling some of these attitudes, for example challenging assumptions around the ethnicity, gender and age of those at risk of an ED.

## Background

Eating disorders (EDs) typically have their onset during adolescence or the transition to adulthood; mean age of onset for anorexia nervosa (AN) and bulimia nervosa (BN) is between 15 and 19 years, although there is greater variability in binge-eating disorder (BED) onset [1–4]. Individuals commonly present to specialist services with considerable duration of untreated ED. A recent systematic review estimated an average of 29.9 months in AN, 53.0 months in BN, 43.8 months in other specified feeding and eating disorders (OSFED) and 67.4 months in BED [5]. Lengthy duration of untreated ED represents a major challenge for successful intervention, as illness duration is a key predictor of outcome in EDs [6, 7]. Minimising duration of untreated ED will be key to designing and implementing more efficacious and cost-effective treatment strategies for EDs, as well as reducing the impact of EDs on developmental milestones.

Efforts to reduce duration of untreated ED have typically focused on minimising the length of waiting time between treatment-seeking and start of specialist evidence-based treatment [8]. In the United Kingdom (UK), First Episode Rapid Early Intervention for Eating Disorders (FREED) was developed as an service model and care pathway for 16 to 25 year olds presenting with a first-episode ED of less than 3 years duration (“recent onset”) [9]. FREED aims to reduce waiting time between treatment-seeking and start of specialist evidence-based treatment, whilst also adapting said treatment to the specific developmental needs of emerging adults [9]. FREED has been shown to reduce duration of untreated ED and improve clinical outcomes compared to treatment as usual [8, 10–12]. However, whilst initiatives like FREED address service-related delays in ED treatment, duration of untreated ED also encompasses patient-related delays in treatment-seeking [13]. If strategies for early intervention for EDs are to be improved, a clear understanding of the patient-related component of duration of untreated ED is needed [9].

One systematic review synthesised existing research into delays in ED-related help-seeking [14]. It found that most studies have been conducted in community samples of people with a past or current ED diagnosis. Stigma, denial or failure to perceive the severity of the illness, fear of change and practical obstacles to accessing care (e.g. cost, time) were the most commonly experienced barriers. Additional barriers such as lack of support from others, low mental health literacy, preference for self-sufficiency and perceived ineffectiveness of professional help were also identified. Co-occurring mental or physical health problems was the most common facilitator of ED-related help-seeking, followed by concerns about body weight [14]. Accordingly, efforts to increase help-seeking for EDs have typically focused on minimisation of stigma and addressing practical barriers to accessing care [15].

If strategies to hasten professional help-seeking are to be maximally effective, research must go beyond documenting generic reasons for delay and identify specific sources of delay across different ED diagnoses and populations [5]. There are strong indications that the transition to adulthood represents a risk-period for delayed (or absent) help-seeking for mental health problems, including EDs. Several cross-sectional and longitudinal cohort studies have found that individuals in their late teens and early twenties are less likely to seek help and use mental health services than both adults above 25 years and adolescents, despite high rates of mental health difficulties [16–18]. Whilst there are comparatively few studies of help-seeking in EDs, there are indications that up to 80% of university students with clinically significant eating difficulties report not seeking help for these [19]. Additionally, several studies have found that emerging adults (~ 18–25 year olds) who do come to the attention of specialist services have longer duration of untreated ED compared to adolescents [20, 21].

Studies have found that stigma, self-sufficiency and concerns about confidentiality are predominant barriers to mental health-related help-seeking during transition to adulthood [22–27]. Living independently of parents also appears to be associated with decreased likelihood of mental health-related help-seeking during this time [17]. However, scarce research has focused specifically on reasons for delayed ED help-seeking during transition to adulthood. Some studies have examined ED help-seeking in university students, and have found that low perceived need, practical considerations and preference for self-sufficiency are important barriers in university student populations, yet such findings cannot be easily extrapolated to non-university attending young people [15, 28, 29].

There has been a considerable shift in recent years in how the transition to adulthood is understood, yet these new conceptualisations have not been reflected within the help-seeking literature [30]. There has been a growing consensus amongst researchers that adulthood is not achieved until the mid-twenties [30–32]. Some researchers have suggested that, in Western cultures, the period between when a person leaves secondary school and when they attain adult roles (~ 18–25 years) should be considered as a stand-alone developmental stage called “emerging adulthood” [33]. Whilst there is some overlap with adolescence in terms of developmental tasks (e.g. identity and autonomy development), emerging adulthood is understood to be associated with a social context and pattern of psychological characteristics and brain development distinct from both adolescence and subsequent adulthood [33]. Ongoing physical, psychological and social development may contribute to delayed ED-related help-seeking during emerging adulthood [34].

Some research has been conducted exploring ED aetiology and treatment within the context of emerging adulthood [34]. However, just two studies to our knowledge have explored barriers and facilitators of help-seeking for EDs during emerging adulthood. A recent quantitative study was conducted in a community sample of Australian emerging adults with subclinical ED symptoms or past / current ED diagnosis or treatment. It identified denial and fear of change, stigma and self-sufficiency as key barriers to ED help-seeking, providing further evidence that previously identified factors are also relevant to this life-stage [35]. One qualitative study focused on a community sample of Asian American emerging adults with body image concerns and found that available resources and familial support were important facilitators of help-seeking. It also found that stigma was a major barrier to accessing care [36].

No studies to date have focused on understanding factors specific to emerging adulthood or been explicitly informed by current conceptualisations of the unique characteristics of emerging adulthood. Furthermore, existing studies have included heterogenous samples recruited from the community. Developing help-seeking interventions which work in tandem with FREED and other similar early intervention care pathways will require understanding of the barriers and facilitators specific to the target population (i.e. emerging adults with first episode, recent-onset EDs).

### Study aim

This study aimed to explore attitudes towards ED symptoms, and their implications for help-seeking, amongst emerging adults receiving treatment for an ED through an early intervention care pathway.

## Method

The study used a qualitative methodology which is well-suited to facilitating nuanced understandings of the complexities of help-seeing during emerging adulthood. The study design was informed by a critical realist philosophical framework [37, 38]. Research informed by this philosophy attempts to go beyond empirical description of social phenomena, instead focusing on identifying causal mechanisms underpinning such phenomena. This makes critical realism particularly well-suited to phenomena that are deemed problematic (e.g. delayed help-seeking), as increased understanding of causal mechanisms can suggest targets for intervention.

### Participants

Participants were drawn from the FREED-Up study, which focused on the continued evaluation of the effectiveness and scalability of the FREED service model (see [9, 13] for further details). The FREED-Up study included 278 emerging adults (18–25 years old) with first-episode, recent-onset (< 3 years) (as ascertained by a structured onset interview) DSM-5 EDs (AN, BN, BED or OSFED) who presented for treatment at four specialist ED services in England between 2016 and 2018. The present study specifically focused on exploring emerging adults’ attitudes towards their ED symptoms and help-seeking. Selected FREED-Up study participants were informed of this additional study by phone or email by one researcher (RP). Potential participants were told that the study was about their experiences of getting help and receiving treatment for their eating difficulties. No time-limit was placed on consideration of participation. A purposive sampling strategy was used to explore a range of attitudes and experiences. Participants were recruited according to characteristics of putative relevance to the research question (as suggested by existing research evidence) (e.g. age at assessment; diagnosis; gender; ethnicity; living situation; geographic location). In practice, the sampling strategy involved the researcher accessing existing FREED-Up demographic and clinical data and approaching participants accordingly. No monetary incentive was offered for participation. Estimated sample size was informed by the approximate number of participants required in previous comparable studies [28, 39]. However, recruitment continued until saturation; that is, until the devised themes had been fully explored and new data were easily accommodated within them. In total, 45 emerging adults were approached to participate to reach the final sample size of 14 (31%).

### Procedure

Ethical approval for the FREED-Up study was granted by the Camberwell St. Giles Research Ethics Committee (ref: 16/LO/1882) and NHS Health Research Authority. All FREED-Up study participants who received the FREED intervention (*N* = 278) completed detailed online questionnaires upon entry to the study (i.e. after first specialist contact). These included demographic data and measures of ED symptoms and duration of ED (for further details see [13]). Semi-structured interviews for this study were conducted on a one-to-one basis by RP (a researcher not involved in treatment), either over the phone (71%) or in person. Care was taken to establish rapport and promote open discussion over the phone (e.g. ensuring the participant was in a private location). Interviews were structured around a topic guide informed by evidence-based conceptualisations of both ED-related help-seeking and emerging adulthood (see Appendix for interview topic guide). Participants were asked to narrate their journey from experiencing changes in eating or body image-related thoughts or behaviours to accessing specialist treatment, and to identify any particular barriers or facilitators to help-seeking. Questions also reflected awareness of several specific characteristics of emerging adulthood: i) increasing independence from parents; ii) transitions and instability; iii) ongoing identity exploration, and sought to identify any relationship between such characteristics and help-seeking. The topic guide was used flexibly and revised iteratively. The average length of the interview was 33 min. All interview audio was recorded and transcribed verbatim. All identifying information was removed at point of transcription. Participants were assigned pseudonyms.

### Data analysis

Quantitative data was analysed using SPSS software, version 26. Qualitative data analysis was undertaken concurrently with data collection, using NVivo software, version 10. Data analysis used the six steps of thematic analysis outlined by Braun and Clarke (2006) (see Table 1). Consistent with the critical realist framework, the study used a primarily deductive yet flexible coding process. Coding was therefore informed but not constrained by existing theory and literature on emerging adulthood and ED-related help-seeking [37]. Steps three, four and five of the thematic analysis process focused on exploring latent themes and causal mechanisms underlying emerging adults’ descriptions of their help-seeking [37]. The dataset was repeatedly reviewed and discussed by the authors to ensure the emerging themes fit with the original data.

**Table 1:** Steps of thematic analysis

**Step**	**Process**
**1.** **Familiarization with data**	Transcribe; read and re-read data-set; note down initial ideas
**2.** **Generation of initial codes**	Code data-set
**3.** **Search for themes**	Collate codes into potential themes
**4.** **Review themes**	Check themes against coded extracts and the entire data set; generate a thematic map
**5.** **Define and name themes**	Refine the specifics of each theme; generate clear names and definitions
**6.** **Write up**	Select and analyse quotations; write a report of the analysis

## Results

### Participant characteristics

The final study sample consisted of 14 participants (see Table 2 for summary of participant characteristics, compared to those who declined participation). As noted, all participants had received (*N* = 5; 35.7%) or were receiving (*N* = 9, 64.3%) specialist outpatient or day-patient ED treatment in one of the four participating ED services.

**Table 2:** Participant Demographics

	Interviewed (*N* = 14)	Declined / DNR to Interview Request (*N* = 31)
Age (at first specialist contact)	*M =* 20.86 (SD = 1.99)	20.16 (2.46)
DUED (months)	*M =* 21.50 (SD = 10.55)	19.37 (9.42)
BMI (at first specialist contact)	*M =* 18.87 (SD = 3.23)	20.47 (5.23)
EDE-Q total score (at first specialist contact)	*M =* 4.53 (SD = 0.72)*	3.78 (1.23)*
Gender	93% female (*N* = 13)	94% female (*N* = 29)
Eating Disorder Diagnosis		
AN	5 (35.71%)	11 (35.48%)
BN	5 (35.71%)	10 (32.26%)
BED	1 (7.14%)	1 (3.23%)
OSFED	3 (21.43%)	9 (29.03%)
Ethnicity		
White	10 (71.43%)	18 (58.06%)
Asian	0 (0%)	3 (9.68%)
Black	0 (0%)	2 (6.45%)
Mixed	2 (14.29%)	3 (9.68%)
Unspecified	2 (14.29%)	5 (16.13%)
Occupation		
Student	7 (50.0%)	17 (54.83%)
Employed	7 (50.0%)	10 (32.26%)
Unemployed	0 (0%)	2 (6.45%)
Unknown	0 (0%)	2 (6.45%)
Living Situation		
With family	5 (35.71%)	19 (61.29%)
With peers	7 (50.0%)	8 (25.80%)
With partner	0 (0%)	2 (6.45%)
Alone	0 (0%)	2 (6.45%)
Other / Unspecified	2 (14.29%)	0 (0%)

* These figures were significantly different at *p* < 0.05

*Abbreviations: AN* anorexia nervosa, *BMI* body mass index, *BN* bulimia nervosa, *DNR* did not respond, *DUED* duration of untreated eating disorder, *EDE-Q* Eating Disorder Examination Questionnaire, *OSFED* other specified feeding or eating disorder, *M* mean, *SD* standard deviation

### Thematic analysis

**Fig. 1:**
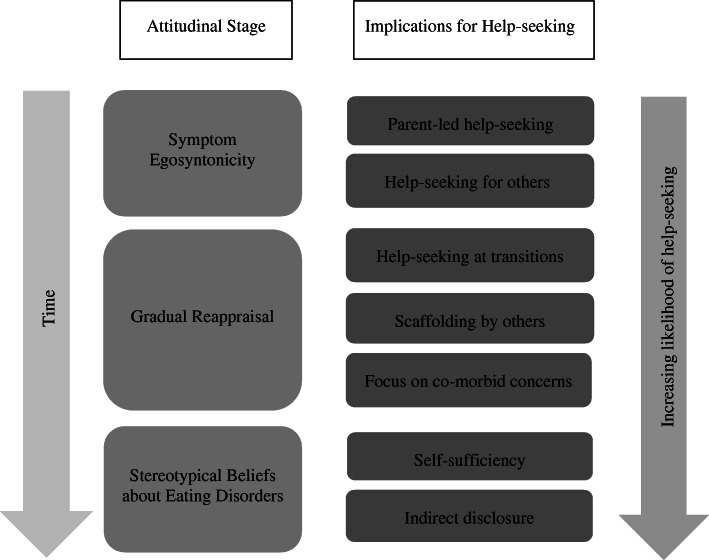
Attitudinal stages and their implications for help-seeking

Three key themes relating to participants’ attitudes towards their ED symptoms prior to help-seeking were identified in the data: 1. Symptom egosyntonicity; 2. Gradual reappraisal; 3. Feelings of exclusion from ED discourse. There was a clear temporal dimension to these attitudes, such that participants tended to move through these phases in the order presented. Each attitude was associated with distinctive implications for help-seeking (see Fig. 1 for summary). The data was examined for differences according to participant characteristics (e.g. diagnosis; stage of treatment), but these did not appear to be associated with differences in the themes identified.

#### Phase 1: symptom egosyntonicity

The majority of participants described that increased control over what, how much or how often they ate was the first change in their eating-related thoughts and behaviours they could recall. All participants who experienced increased dietary control (and resultant weight loss) described how this was not perceived as a problem, but rather was highly egosyntonic (i.e. positively appraised and valued).

*“I just thought I was doing a good thing, sort of exercising more, trying to be healthy, trying to lose a bit of weight.”* (Emily, 19 years old, AN)

Several sub-ordinate themes appeared to contribute to this initially positive appraisal.

##### Self-esteem

For most participants, increased dietary control and weight loss in the early stages gave them a sense of achievement and bolstered their self-esteem. This increased confidence was further strengthened by positive feedback on their dietary control and/or weight loss from friends and family.

*“It gave me a sense of achievement and I felt good about myself.”* (Christina, 19 years old, AN)

##### Coping with life-stress

Almost all participants reported experiencing stressful life-events (e.g. relationship break-ups, university exams, moving to a new city). Several participants reported that dietary control provided a sense of control which they felt they lacked in other aspects of their life, therefore helping them cope with these stressful situations.

*“As I got more stressed [eating] became the control mechanism. [ …*] *I didn’t necessarily see it as a problem at that point. It was a way of dealing with the anxiety.”* (Emily, 19 years old, AN)

#### Implications for help-seeking

Several participants described how their positive appraisal of their symptoms in the early phases meant they lacked motivation to seek help. When it did occur, help-seeking had several distinctive characteristics.

##### Parent-led help-seeking

One participant (Emily, 18 years old, living with her parents) described how, early in her ED, she was resistant to seeking help. However, her mother sought help on her behalf, bringing up her concerns in an appointment with a general practitioner (GP) for an unrelated health issue.

*“[my mum] said “I’m really concerned about her weight and what she’s eating, it doesn’t seem right”, and then the doctor sort of grilled me on how I was eating … I was really angry [with her].”* (Emily, 19 years old, AN)

##### Help-seeking for others

Several participants described how, early in their ED, friends (and sometimes family) raised concerns about changes in their eating behaviour or their weight loss and suggested they should seek support. In some cases, participants described being resistant to accepting help, but going through the motions of professional help-seeking to placate friends or family.

“*I think that I went [to the GP] kind of to appease [my flatmate]”* (Gemma, 20 years old, OSFED)

#### Phase 2: gradual reappraisal

The majority of participants described that, whilst initially they interpreted their change in eating-related thoughts and behaviours positively, this appraisal changed over time. Several sub-ordinate themes appeared to contribute to this gradual lowering of their initially positive appraisal of these symptoms.

##### Binge-eating and compensatory behaviours

Several participants reported that over time they started to experience bingeing and compensatory behaviours. These behaviours lacked the social desirability of dietary control and were therefore more negatively appraised.

*“I became more understanding of [my condition] with the bingeing, [whereas] with the restricting I felt like I was just following a healthy diet.”* (Charlotte, 21 years old, BN)

#### Compulsivity

The majority of participants described how over time their ED behaviours started to feel less under their control, and more automatic and compulsive. Several participants reported that stressful life-events (e.g. relationship break-ups; moving home to parents) were associated with increased compulsivity.

*“[At the beginning] it felt like a choice rather than [...] now, where I can’t help it.”* (Gemma, 20 years old, OSFED)

#### Physical and mental health

Many participants described how they started to feel the impact of their symptoms on their physical and mental health (e.g. fatigue; muscle pain; low mood; anxiety).

*“I could feel the physical effects [of bingeing and purging] and I started to get worried about that … My hands were shaking quite often … I’d noticed blood in my vomit … my gums were bleeding a lot.”* (Sasha, 20 years old, BN)

#### Social functioning

Several participants identified that symptoms began to negatively impact many life domains (e.g. social life; career; family life).

*“After a couple of months, it starts affecting everything else, you withdraw from people and other things in your life [ …*]. *I think it was then when I started thinking this really isn’t great.”* (Max, 24 years old, OSFED)

For some participants, impact was conveyed via comparison of their own experiences of their peers and what they perceived as “normal”. For several participants, they became particularly aware of the impact on their functioning following a big life-change (e.g. moving to a new city; starting university), when they found themselves unable to function in the way they would like to (e.g. making new friends; being fully engaged in their course). However, the gradual nature of this reappraisal (and ongoing shame and embarrassment about symptoms) was such that many participants experienced a period of uncertainty about whether they had an ED or not, and/or ambivalence towards the idea of seeking and receiving help.

*“I was sort of seeing some of the patterns and thinking that’s not quite right, but I don’t really believe what it is, and I don’t really understand what it is.”* (Max, 24 years old, OSFED)

#### Implications for help-seeking

This attitudinal phase had several important implications for help-seeking. Help-seeking was more likely during this phase than in the previous phase; however, it was on a knife-edge (i.e. as likely to happen as not).

#### Help-seeking at transition points

Several participants reported that changes in their life situation (e.g. starting university or a new job; moving to a new city) were associated with symptom reappraisal. Some participants wanted to change their symptoms but did not seek professional help because they hoped that this “fresh start” or “change of scene” would be sufficient help in itself, thereby forgoing the embarrassment associated with help-seeking.

*“I thought like new job, I’m in a new place as well, [I can cope on my own].”* (Niamh, 23 years old, BN)

Other participants described how a life-event did prompt professional help-seeking, often because symptoms had worsened or were preventing them from making the most of their new situation.

*“The change of location … was what [pushed me to seek help], because it was sort of the idea of a fresh start [ …*.] *I wanted to make the most of being in London.”* (Emily, 19 years old, AN)

#### Scaffolding by others

Many participants described focusing on their family’s and friends’ responses to their symptoms to help them decide whether to seek help. For many participants, friends and family not raising concerns about their symptoms, or doing so in a vague or generic way (e.g. “is everything OK?”), were interpreted as disconfirming their suspicions that they might have an ED. Such interventions therefore did not bolster their confidence in seeking help, and in fact increased their belief that such problems were shameful or embarrassing.

*“I felt it was kind of just brushed under the carpet. To me it was like, maybe I don’t have an eating disorder [ …*]. *It just made me ignore it even more.”* (Christina, 19 years old, AN)

In contrast, several participants described how detailed enquiries about wellbeing or explicit statements of concern often prompted help-seeking. Several participants described how others assuming some of the responsibility for help-seeking and providing practical assistance (e.g. registering for the doctor; scheduling appointments; accompanying the participant to appointments) was intrinsic to successful help-seeking whilst they felt uncertain and ambivalent.

*“My friend messaged me like “I think you need to go to the doctors”. She came with me to the doctors to sign up, because I wasn’t even registered … she came to all my doctor’s appointments with me.”* (Olivia, 21 years old, BN)

#### Help-seeking for co-morbid concerns

Several participants described how seeking help for issues related to their ED symptoms (e.g. low mood; muscular pain), whilst also highly resistant to addressing their eating problems.

*“I felt the physical effects but emotionally I felt different. I didn’t really want to stop.”* (Sasha, 20 years old, BN)

### Phase 3: feelings of exclusion from eating disorder discourse

Following a period of gradual reappraisal, the majority of participants described coming to a point of problem awareness i.e. where they perceived their symptoms as a problem for which they needed help. However, participants also reported awareness of dominant constructions of EDs. When participants felt they did not fit within this discourse, they believed that asking for help would be embarrassing and shameful, and their concerns would be rejected. Two key exclusionary aspects of discourse were identified as follows:

#### “You have to be extremely thin to have an eating disorder”

Many participants expressed the belief that EDs are characterised by extreme low weight. They described how they did not see themselves as being sufficiently low weight to have their concerns accepted, both by friends and family and by key professionals.

*“I was afraid they wouldn’t see me as ill. I thought that if I told any of my friends, they would just laugh in my face and tell me “that’s not true””.* (Gaia, 22 years old, AN)

#### “Eating disorders are teenage illnesses”

Several participants described beliefs that EDs were *“immature”* and *“childish”* (Charlotte, 21 years old) illnesses, commonly experienced by teenagers. Participants anticipated having their concerns rejected by others because they were not teenagers.

*“I am 22 [ …*], *I’m not 16. How did I get into this? Why am I not able to snap out of it? It’s such a stupid issue, and I’m just ashamed of it.”* (Gaia, 22 years old AN)

#### Implications for help-seeking

This attitudinal phase had several important implications of help-seeking. Whilst help-seeking during this phase was more likely than in previous phases, the majority of participants reported that feeling excluded from discourse about EDs - and associated shame and embarrassment - impacted help-seeking. When help-seeking did occur, it was typically orchestrated in a manner designed to minimize embarrassment. Accordingly, it had several distinctive characteristics.

#### Preference for self-sufficiency

Several participants described how feeling excluded from ED discourse and ensuing concerns about embarrassment were associated with them preferring to cope with their problems alone.

“*I was a bit embarrassed or cautious about telling my family. I tried very much to handle it by myself.”* (Charlotte, 21 years old, BED)

Several participants described circumnavigating help-seeking from family and friends or key professionals, choosing to cope with their problems alone.

#### Utilisation of self-help resources

Several participants described turning to self-help resources to avoid the embarrassment associated with help-seeking. Participants noted that, whilst such resources often did little to alleviate symptoms, they did increase their confidence that their concerns would be taken seriously. It appeared that resources which incorporated other (non-adolescent) people’s experiences of EDs were particularly impactful, and helped participants feel less embarrassed.

#### Seeking help independently of parents

Several participants described considering accessing professional help independently of their parents’ involvement to avoid embarrassment. However, participants perceived it as difficult to do so. Several participants described how they did not go to key professionals (e.g. GP: teachers), for fear any information confided would be shared with their parents. Several participants did seek professional help following assurances (e.g. online research, verbal confirmation from GP that details would not be shared with their parents). However, in one case a participant’s fears of parental involvement was realised (i.e. automated appointment reminder was sent to parents’ contact details).

#### Indirect disclosure of difficulties

The majority of participants described that feeling excluded from ED discourse and resultant fear of rejection and embarrassment meant they found it difficult to communicate their difficulties, to both close others and key professionals.

*“I just couldn’t speak about it [with the GP] because [ …*] *it’s embarrassing, and it’s just, I didn’t really know what to say?”* (Olivia, 21 years old, BN)

Several participants described how this meant they sought alternative, indirect ways to communicate their need for help.

##### Written communication

Several participants described how communicating their difficulties face to face was associated with a particularly high potential for embarrassment. For many participants, using a written form of communication (e.g. social media post; email to university counselling service; university registration form) was integral to their help-seeking.

##### Generic communication of distress

Several participants described the prospect of using the term “eating disorder” or specific diagnoses (e.g. AN; BN), or outlining in specific detail their eating or compensatory behaviours, as particularly embarrassing. For many participants it appeared important to be able to express their difficulties in vague or ambiguous way (e.g. using non-specific descriptions; crying) and still be understood and taken seriously.

##### Physical health concerns

In contrast to the non-specific approach previously described, several participants described focusing on their physical health concerns to communicate their difficulties in a way that they deemed likely to be acceptable and least embarrassing. Participants described scheduling appointments with healthcare professionals ostensibly for physical health concerns (e.g. medication review with psychiatrist; blood test results from GP), even if their hope was to receive help for their eating problems.

## Discussion

### Summary of findings

This study is one of the first to explore help-seeking for EDs amongst emerging adults specifically. Symptom egosyntonicity, gradual reappraisal and feelings of exclusion form ED discourse were identified as key attitudes relevant to help-seeking amongst emerging adults. There was a clear temporal dimension to these attitudes (although backward movement was possible), such that early in illness symptoms tended to be highly egosyntonic but were gradually reappraised over time. Later, emerging adults perceived their symptoms as a problem but feelings of exclusion from ED discourse and resultant shame and embarrassment became prominent. Each of these attitudes had distinctive implications for whether - and how - emerging adults sought help for their eating difficulties.

#### Phase 1: symptom egosyntonicity

The present study found that emerging adults experienced symptom egosyntonicity in the early phases of ED. Dietary control and consequent weight loss were positively appraised and perceived to boost confidence and help cope with stressful situations. This is consistent with previous research, which has identified egosyntonicity as an important clinical characteristic of AN-type EDs in particular [40, 41]. Whilst egosyntonicity might be common across age-groups, the perceived “coping with life-stress” function of EDs may be particularly pertinent to emerging adults. Stressful life events are a core characteristic of emerging adulthood, and are experienced in greater numbers by emerging adults, than by older age-groups [42]. Indeed, a recent study which examined university students’ experience of EDs, found that participants understood ED behaviours as a way to manage external stressors [43]. Consistent with previous studies across age-groups, this study found help-seeking is unlikely when symptoms are highly egosyntonic [14]. However, this study outlines more specific implications for help-seeking when egosyntonicity is present during EA. Research in adolescents has found that parental intervention is an important driver of help-seeking for mental health problems [44]. However, in the present study just one participant reported parent-led help-seeking. Interestingly, there was some evidence of help-seeking for others, whereby emerging adults sought help to placate friends and/or family. Increased relevance of peers to the help-seeking process - and movement from enforced to placatory help-seeking in particular - is likely linked with emerging adults’ relative autonomy from their parents, compared to adolescents [45, 46].

#### Phase 2: gradual reappraisal

This study found that, as the ED progresses, symptoms tend to be gradually reappraised. In particular, emerging adults become troubled by the addition of new, less socially desirable symptoms (e.g. bingeing) and increasing compulsivity of existing symptoms. Additionally, emerging adults become concerned about the impact of their symptoms on their mental and physical health, and their ability to live a normal life. During this phase, emerging adults were often deeply ambivalent about receiving help. This ambivalence towards seeking and receiving help is also evident in the wider FREED-UP study where 9% of emerging adults referred for specialist treatment could not be contacted after referral or did not attend their assessment appointment [13].

This study found that gradual reappraisal had several implications for help-seeking. Life-events and transitions often prompted symptom reappraisal amongst emerging adults. However, such transitions were often a double-edged sword for professional help-seeking. Emerging adults sometimes viewed transitions as a “fresh start” (i.e. sufficient help in itself), a determination often underpinned by feelings of shame and embarrassment regarding their experiences. Indeed, this finding echoes that of a recent qualitative study, which found that moving to university specifically was perceived as a “new start” and associated with minimisation of perceived severity of the young person’s ED [43]. Other times transitions were viewed as an impetus for help-seeking. To our knowledge, the role of life-events has not been previously reported in the help-seeking literature. Stressful life-events and transitions may be particularly pertinent amongst emerging adult populations, given that these are a core characteristic of emerging adulthood, and are experienced in greater numbers by emerging adults than by older age-groups [33, 42]. This study found that perceived negative aspects of the illness (e.g. low mood; physical effects) are often the focus of help-seeking efforts, whilst emerging adults may remain reluctant to seek/accept help for other, more valued aspects of the ED. Such findings are consistent with previous research which has highlighted physical and mental health problems as important facilitators of help-seeking [14]. Indeed, in the wider FREED-Up cohort 53% of participants identified non-ED specific concerns – most commonly depression, anxiety, disturbed sleep and academic problems - as the desired focus of their treatment [47].

The findings of the present study indicated that family and friends “turning a blind eye” to ED symptoms was associated with delayed help-seeking. This is consistent with previous research, which has found that lack of support and understanding from family and peers are important barriers to help-seeking for EDs [14]. Conversely, others taking a hands-on approach to help-seeking was often a powerful facilitator. This echoes previous studies of female university students and Asian American emerging adults, which found that familial support and expressions of concern facilitated help-seeking [28, 36]. The important role of assertive intervention by others during this attitudinal phase contrasts with the negative response emerging adults may have to such directive efforts (e.g. parent-led help-seeking) when symptoms are highly egosyntonic. It therefore highlights that attitudinal phase is important, such that emerging adults may have different responses to similar interventions dependent on their attitudinal phase.

#### Phase 3: feeling excluded from eating disorder discourse

This study found that, following a period of reappraisal, emerging adults with EDs tend to come to understand their ED symptoms as a problem. However, they often find it difficult to locate their experiences within dominant discourses about EDs (e.g. that EDs are characterised by extreme low weight), and this is a source of shame and embarrassment. This is consistent with previous studies, which have highlighted that stigma is a predominant barrier to mental health-related help-seeking, and that ED discourses are often experienced as exclusionary [14, 22–27, 35, 36, 48]. In addition to predominant ideas about the relationship between weight and EDs, there is evidence that emerging adults believing they are “too old” for EDs may be a particularly pertinent barrier to help-seeking in this age group. This adds important nuance to existing research and suggests that stigma may manifest differently in distinct population groups.

This study identified several implications of exclusionary ED discourse and resultant shame and embarrassment for help-seeking. Consistent with previous findings, this study indicated that many emerging adults have a preference for self-sufficiency, with many deciding to deal with their difficulties alone [15, 24–27, 35]. This study identified that many emerging adults use self-help resources as a first port of call. Additionally, many emerging adults prefer to access professional help with assurances that parents will not be involved in the process, but this is often difficult. This is consistent with previous studies of mental health related help-seeking during transition to adulthood, which has found that concerns about confidentiality and trust are common barriers during this life-stage [24–27]. Indeed, a subjective sense of being “in-between” adolescence and adulthood is a core characteristic of emerging adulthood, and it therefore makes sense that emerging adults also feel this uncertainty with regard to their right to confidentiality [33].

This study found that emerging adults appeared to find several ways to circumnavigate face-to-face confessionals, including written communication, expressing their distress in generic terms, and focusing on their physical health concerns. The focus on physical health in particular is consistent with anecdotal evidence from ED clinicians, who find that many patients report giving “hints” to their GP over the course of multiple visits [49]. Furthermore, there is evidence from another study that in the 5 years prior to diagnosis people with EDs consult their GPs more than other people, most usually for gastrointestinal, gynaecological and psychological complaints [50].

In summary, many of the previously cited barriers to help-seeking for EDs in general, seem to also apply to this emerging adult population. However, there are some important nuances (e.g. instability, perceptions of them being teenage illnesses, concerns about parental involvement) that are more intense than in adolescent or older adult populations.

### Clinical implications

This research might be used to inform the development and evaluation of interventions which aim to encourage help-seeking amongst emerging adults with first episode, recent onset EDs. In particular, interventions should be informed by knowledge of the attitudinal phase outlined here, seeking to identify attitudinal phase and tailor support and encouragement accordingly. Clinical implications are presented in Table 3.

**Table 3:** Clinical Implications

Predominant attitude towards help-seeking	Implications for help-seeking interventions
**1.** **Symptom egosyntonicity**	Focus on enhancing motivation to seek help (e.g. using motivational interviewing techniques) Support family / friends to raise concerns and encourage placatory help-seeking
**2.** **Gradual reappraisal**	Focus on inter-relatedness of ED and other difficulties (e.g. low mood; physical health) Increase awareness amongst professionals likely to have routine contact during transitions of increased openness to receiving help Streamline registration/appointment-booking processes at likely help-seeking avenues (e.g. GP) Encourage family/ friends to scaffold help-seeking (e.g. booking appointments)
**3.** **Feelings of exclusion from eating disorder discourse**	Focus on mapping negative effects of eating difficulties / clarifying preferred life directions, rather than slotting into diagnostic categories Integrate help-seeking interventions with self-help resources Communicate the importance of confidentiality Facilitate circumnavigation of face-to-face communication (e.g. emails; texts) at likely help-seeking avenues

### Strengths and limitations

Participants in the present study were drawn from a well-established and comprehensive cohort study. Data pertaining to key demographic and clinical characteristics at first specialist contact were available, and therefore provide a clear picture of the characteristics of these participants around the time help-seeking occurred. However, it might also have been informative to collect data on ED symptoms at time of interview to understand how differential treatment response might have impacted participants’ understanding of their help-seeking.

This study used a purposive sampling strategy to explore a range of perspectives and experiences, and therefore did not aim to achieve statistical generalizability. Whilst the characteristics of the sub-sample of the study broadly map onto the FREED-Up cohort as a whole, quantitative research is required to verify the extent to which these findings generalize to emerging adults receiving treatment for first-episode EDs.

Just one-third of individuals approached chose to participate in this study. Given that participants were told that this was a study about their experiences of getting help for eating difficulties, it may be that the participants who decided to participate may have felt they had particularly notable (positive or negative) help-seeking experiences. Comparison of the demographic and clinical characteristics of those who did and did not participate indicates that this self-selecting third are broadly similar to those who decided not to participate on a range of characteristics. Interestingly, whilst both groups had comparable duration of untreated ED, those who agreed to participate in the interview did have less severe ED symptoms at first specialist contact. It is possible that severity of symptoms impacted their help-seeking in a myriad of ways, and different barriers and facilitators may apply to people who seek help when more unwell.

Finally, this study included just one participant with BED and one male participant. Previous research suggests that experiences of help-seeking may vary according to gender and diagnosis [51, 52]. However, this study was unable to examine this in any depth due to a lack of diversity of gender and diagnoses within the sample.

### Future research

Future research might usefully focus on the validation of these findings using quantitative methodologies. For instance, one might systematically assess the impact of life-events on symptom progression and appraisal, and how this relates to help-seeking. Such research might also usefully explore whether such factors are uniquely relevant to emerging adults with first-episode EDs, or also applicable to adolescent and adult populations experiencing EDs for the first time.

Additionally, further studies should aim to investigate experiences of help-seeking amongst male and black and minority ethnic emerging adults, ascertaining the extent to which the attitudes and experience found in this sample also pertain to these groups. It may be that interventions may need to be further tailored to meet the needs of particular sub-groups of emerging adults, addressing the parallel processes of shame and embarrassment experienced by emerging adults and their families.

The present study aimed to understand help-seeking for ED symptoms amongst emerging adults receiving treatment for an ED through an early intervention care pathway. Accordingly, all participants in the present study had received specialist treatment for their ED and had done so relatively early in their illness, with an average duration of untreated ED of 20.14 months, compared to averages between 29.9 to 67.4 months across diagnoses indicated by a previous systematic review [5]. Future research should therefore focus on elucidating the factors that might be relevant for other sub-groups of emerging adults who experience EDs (e.g. emerging adults who seek help much later in their illness, or never do).

Further research is needed into emerging adults’ experiences of interactions with healthcare professionals. Additionally, it will be important to understand the experiences of other stakeholders in the help-seeking process (e.g. healthcare professionals; university staff; friends and family). Studies might usefully identify some of the barriers such stakeholders face in identifying EDs during emerging adulthood and supporting emerging adults to seek help.

## Conclusion

This study identified symptom egosyntonicity, gradual reappraisal and feelings of exclusion for ED discourse as key attitudes relevant to help-seeking amongst emerging adults with first-episode, recent-onset EDs. Each of these attitudes had distinct implications for whether - and how - emerging adults negotiated the need for treatment and engaged in treatment for their eating difficulties (e.g. help-seeking for others; preference for self-sufficiency; indirect disclosure). This research might be used to inform the development and evaluation of interventions which aim to facilitate help-seeking amongst emerging adults with first episode recent onset EDs, and their ongoing engagement with treatment services once sought.

## Data Availability

The datasets used and/or analysed during the current study are available from the corresponding author on reasonable request.

## Appendix

### Interview Topic Guide

1. Can you tell me about when behaviours and thoughts around food / body changed?

**Probes:**

- Changes noticed
- Feelings towards / understanding of changes
- Life context

2. How did things progress?

**Probes:**

- Progression of symptoms
- Changes in feelings / understanding
- Life context

3. Can you tell me about your experience of asking for help for your eating difficulties?

**Probes:**

- When?
- How / what said / who from?
- Why then?
- Life context

4. How would you summarize the reasons for the delay in getting help?
5. Were there any things that helped you to get help?
6. What about family and friends, did they play any role in your help-seeking?

**Probes:**

- Who? When? How? What said?
- Reasons
- Reactions
- Impact on professional help-seeking
- Independence from parents (e.g. access to healthcare)

7. Did you have any big life changes (e.g. moving cities, relationship break ups) between experiencing symptoms and seeking help?
  **Probes**

- Impact on symptoms / help-seeking

8. Did you have any knowledge of eating disorders and who they affect?

**Probes**

- How well do they match with own identities / characteristics?
- Impact on symptoms / help-seeking

## Abbreviations

AN: anorexia nervosa
BED: binge-eating disorder
BN: bulimia nervosa
EDs: eating disorders
FREED: First Episode Rapid Early Intervention for Eating Disorders
OSFED: other specified feeding and eating disorders
UK: United Kingdom
